# Gene editing to enhance biotic stress tolerance in sugarcane

**DOI:** 10.3389/fpls.2025.1750169

**Published:** 2025-12-26

**Authors:** Fredy Altpeter

**Affiliations:** 1 Agronomy Department, Plant Molecular and Cellular Biology Program, Genetics Institute, University of Florida, Institute of Food and Agricultural Sciences (IFAS), Gainesville, FL, United States; 2 DOE Center for Advanced Bioenergy and Bioproducts Innovation, Gainesville FL, United States

**Keywords:** gene editing, CRISPR-Cas systems, TALEN, disease resistance, insect resistance, herbicide resistance, bioenergy, sugarcane

## Introduction

Sugarcane (*Saccharum* spp.) contributes approximately 80% of global sugar production and 40% of biofuel while serving as a promising feedstock for bioproducts (Brant et al., 2025). However, productivity faces mounting challenges from biotic threats including fungal, viral and bacterial diseases (Rott, 2018). Traditional breeding approaches are severely constrained by sugarcane’s complex polyploid genome containing 10–12 copies of "hom(oe)ologous genes within an approximately 10 Gb genome (Healey et al., 2024), extending breeding cycles to 12–15 years. Genome editing has revolutionized crop improvement by enabling targeted modifications without necessarily introducing foreign DNA, potentially circumventing regulatory hurdles while accelerating variety development (Li et al., 2022a). For sugarcane, these technologies offer unprecedented opportunities to enhance biotic stress resilience while improving yield and quality. This article examines current progress and prospects for developing biotic stress-tolerant sugarcane through gene editing, emphasizing technical advances, promising gene targets, and strategic approaches for trait stacking.

## Challenges and opportunities of genome editing in highly polyploid sugarcane

The highly polyploid nature of sugarcane (2n = 10-13x = 100-130) presents distinct challenges for genome editing compared to diploid crops. May et al. (2023) comprehensively outlined these obstacles. The large genome size (~10 GB) and presence of multiple hom(oe)ologous gene copies create several technical hurdles.

The highly complex and repetitive nature of polyploid genomes poses significant challenges for computational biology in terms of phasing, annotation, full chromosome assembly, and differentiation between homologs and homoeologs during generation of polyploid genome sequences (Kyriakidou et al., 2018). Chromosomal rearrangements and epigenetic shifts during polyploid evolution have led to global transcriptome changes including activation of transposable elements and biased expression of homoeologs (Wendel et al., 2018), presenting difficulties for determining chromosomal targets.

All or a larger number of functionally redundant alleles must be successfully co-edited to generate a “loss-off function” phenotype in polyploids. Efficient co-editing requires refined genome editing reagents and protocols. Designing guide RNAs (gRNAs) that target conserved sequences across multiple hom(oe)ologs while minimizing off-target effects requires careful computational analysis. The recent availability of chromosome-level genome assemblies (Healey et al., 2024) has significantly improved our capacity to design specific gRNAs.

Molecular characterization of edited events in polyploid sugarcane requires evaluation of the co-editing of the target gene copies/alleles in multiple tillers to confirm faithful transmission of the edits to vegetative progenies. Brant et al. (2024a) demonstrated that capillary electrophoresis provides similar information content to next-generation sequencing for detecting indels at a fraction of costs. Third generation long-read sequencing technologies including Pacific Biosciences Single Molecule Real-Time (SMRT) and Oxford Nanopore sequencing enable quantification of numbers of mutated copies/alleles in polyploid crops (Curtin et al., 2021).

Vegetative propagation is required to maintain the performance of elite sugarcane cultivars, precluding transgene removal through Mendelian segregation. More efficient, higher-throughput, and non-integrative transformation platforms accelerate development and commercialization of edited polyploid crop cultivars (Altpeter et al., 2016; Gordon-Kamm et al., 2019). While multi-allelic editing poses challenges, the larger numbers of gene copies in polyploid genomes offer opportunities for generation of a range of phenotypes by targeting partial loss of gene expression (Eid et al., 2021; Brant et al., 2024b).

## Gene editing technology: advances in sugarcane

Jung and Altpeter (2016) pioneered targeted genome modification using transcription activator-like effector nucleases (TALENs) to edit the caffeic acid O-methyltransferase *(COMT)* gene, modifying lignin composition and reducing lignin content. Sugarcane lines with co-editing of more than 100 *COMT* gene copies/alleles maintained agronomic performance under field conditions while improving saccharification efficiency for bioethanol production by 44% (Kannan et al., 2018). Ko et al. (2018) demonstrated that this *COMT*-edited sugarcane enhanced bioethanol production by 148% when combined with xylose-fermenting yeast strains.

Eid et al. (2021) validated CRISPR/Cas9 for multiallelic editing by targeting the magnesium chelatase gene, producing chlorophyll-deficient phenotypes confirming successful editing across multiple alleles. Zhao et al., 2021 demonstrated that a short region of homology (30nt) is sufficient for error-free homology directed repair of Cas9 DNA breaks. Efficient and reproducible gene targeting was reported when longer templates with 447 to 1,007 bp homology arms were co-delivered with Cas9. This resulted in precision nucleotide substitutions in several acetolactate synthase gene copies/alleles, conferring resistance to herbicides nicosulfuron and imazapyr (Oz et al., 2021).

Brant et al. (2024b) reported the first field trial of CRISPR-edited sugarcane. Comparing events with different numbers of co-edited copies/alleles of the *LIGULELESS1* allowed to optimize leaf angle for improved biomass yield.

Co-editing challenges arising from multiple gene copies or alleles were addressed through systematic optimization of editing tool expression. Key improvements included codon optimization, intron incorporation, utilization of strong promoters and terminators, and biolistic delivery of minimal expression cassettes. To facilitate identification of events achieving the desired co-editing efficiency across copies and alleles, capillary electrophoresis was employed for rapid, low-cost analysis (Brant et al., 2024a, b).

## Candidate genes for biotic stress tolerance

### Disease resistance

Disrupting host susceptibility genes that pathogens exploit for successful infection via gene editing represents a strategic approach to achieving stable disease resistance in plants (Zaidi et al., 2018). Sugarcane smut (*Sporisorium scitamineum*) is a major fungal pathogen of sugarcane (Rott, 2018). Wang et al. (2025) recently demonstrated that *ScWRKY2* negatively regulates smut resistance through dual mechanisms (transcriptional repression of *ScLRR-RLK* and interaction with ScPsbP), with overexpression increasing susceptibility while resistant cultivars show reduced expression. Therefore, *ScWRKY2* represents a promising knockout target to improve smut resistance.

Targeted mutagenesis of the susceptibility allele *TaLr34* encoding an ATP-binding cassette (ABC) transporter within the ABCG subfamily enhanced resistance in wheat against *Puccinia triticina*, the pathogen causing leaf rust without yield penalty (Javaid et al., 2025). A similar approach targeting *Lr34* homologs in sugarcane could be explored for enhancing resistance to orange rust (*Puccinia kuehnii*) and/or brown rust (*Puccinia melanocephala*).

Leucine-rich repeat receptor-like kinases (LRR-RLK) represent a large gene family serving as pattern recognition receptors involved in regulating stress responses, plant growth and development, and signal transduction (Cheng et al., 2021). Prime editing and base editing technologies, offer precision beyond conventional mutagenesis with CRISPR/Cas9, and still need to be optimized for efficient and precise editing in sugarcane (Li et al., 2024a). Precision editing of LRR-RLK could enhance pathogen-associated molecular pattern recognition.

Sugarcane mosaic disease is caused by potyviruses, including sugarcane mosaic virus, sorghum mosaic virus, and sugarcane streak mosaic virus and is one of the most widely recorded sugarcane diseases, causing severe economic losses. Potyviruses require eukaryotic translation initiation factor 4E (eIF4E) and its iso form (eIF(iso)4E) for infection via viral genome-linked protein (VPg-eIF4E) interactions. Mutating one of these genes can provide virus resistance with minimal pleiotropic effects (Charron et al., 2008).

CRISPR/Cas13-based approaches targeting viral RNA could provide inducible resistance activating only upon infection (Hussain et al., 2024). For DNA viruses like Sugarcane bacilliform virus, CRISPR/Cas9 can directly target and disrupt viral sequences as demonstrated by Tripathi et al. (2019) in banana.

Editing of *SWEET* gene promoters in rice successfully conferred resistance to bacterial blight by preventing *Xanthomonas oryzae* from hijacking sugar transporters through transcription activator-like effector (TALE)-mediated activation via Type III secretion system (T3SS) (Oliva et al., 2019). Conversely, *Xanthomonas albilineans*, which causes sugarcane leaf scald, lacks both TALEs and T3SS and instead employs albicidin, a potent DNA gyrase inhibitor that blocks chloroplast differentiation for pathogenicity (Li et al., 2022b). Since chloroplastic DNA gyrase is nucleus-encoded, precision editing of the albicidin binding site may enhance sugarcane leaf scald resistance.

### Herbicide resistance

Oz et al. (2021) successfully demonstrated CRISPR/Cas9-mediated multi-allelic gene targeting of acetolactate synthase (*ALS*) genes. By introducing specific point mutations (W574L and S653I) through template-mediated homology-directed repair, they generated sugarcane plants resistant to sulfonylurea and imidazolinone herbicides. This approach illustrates how gene targeting can convert inferior alleles to superior ones. Additional target genes and approaches for gene editing to confer herbicide resistance are reviewed by Sreekanth et al. (2025).

### Insect resistance

While underexplored, CRISPR-based plant genome editing shows promise for pest control by modifying genes that influence insect behavior. Editing rice’s CYP71A1 gene enhanced resistance to striped stem borers and brown planthoppers (Lu et al., 2018). Since insects use volatile compounds, physical traits, and visual cues to locate hosts (Larsson et al., 2004), and specific volatiles can attract beneficial predators (Ayelo et al., 2021), genome editing could manipulate these profiles for pest management while protecting beneficial species.

Gene editing and gene drive strategies to reverse the development of insecticide resistance in pests are also being developed. This involves using CRISPR/Cas9 technology in insects to replace resistance-conferring genes with their original, susceptible counterparts, or to disrupt genes critical to resistance mechanisms (Xu et al., 2025).

## Accelerating target discovery and prioritization of targets

Historically, RNAi experiments have identified promising gene editing targets and determined required suppression levels for desired phenotypes. Field trials showed RNAi suppression of lignin biosynthesis genes reduced lignin 12-16.5% while improving saccharification 28-76% (Jung et al., 2012, 2013, 2016), directly informing successful gene editing campaigns targeting identical pathways (Jung and Altpeter, 2016; Kannan et al., 2018). Tissue culture-independent technologies like virus-induced gene silencing, spray-induced RNAi (Hoang et al., 2022), and virus-induced gene editing (Baysal et al., 2024) still need to be optimized/developed for sugarcane and will enable rapid phenotypic evaluation of candidate genes before commencing extensive stable transformation/editing projects.

Given the abundance of biotic stress-responsive candidates, systematic prioritization maximizes success. Immediate targets include negative regulators with demonstrated loss-of-function benefits like *ScWRKY2* for smut resistance (Wang et al., 2025). Promising candidates can be prioritized through VIGS/SIGS. Priority should be given to knockout targets creating transgene-free products eligible for regulatory exemptions in jurisdictions like Argentina, Brazil, India, and Australia (Schmidt et al., 2020).

Multiplexed editing will enable simultaneous modification of multiple genes/traits. Orthogonal synthetic transcription factors enable simultaneous activation and repression using orthogonal dCas proteins fused to transcriptional activators and repressors (Pan et al., 2021; Vazquez-Vilar et al., 2023), allowing upregulation of protective genes while suppressing negative regulators. While powerful for altering complex transcriptional networks, this approach will require continuous expression of the synthetic transcription factors and therefore will not be exempt from GMO regulation in most regulatory frameworks.

## Advances in genetic transformation and precision editing

Side-by-side comparison of biolistic and Agrobacterium-mediated transformation revealed both approaches deliver quality events with similar efficiency (Jackson et al, 2013; Joyce et al., 2013; Wu et al., 2015). Morphogenic genes (*BBM*, *WUS2*) have great potential to overcome genotype recalcitrance (Lowe et al., 2016). Ternary vector systems with additional virulence genes demonstrated transformation efficiency increase in maize and sorghum (Anand et al., 2018; Li et al., 2024b). Base editing and prime editing enable specific nucleotide changes without double-strand breaks and random integration of templates (Molla et al., 2021). These technologies still need to be optimized for efficient editing in sugarcane and will allow upregulation of resistance genes by targeting promoter regions, alternative start codons or micro-RNA binding sites (Molla et al., 2021). They will also enable precision edits of herbicide or toxin binding sites, conferring herbicide or pathogen resistance, respectively. Synthetic *in planta* directed evolution combining iterative editing cycles with selection could leverage sugarcane’s genetic redundancy to develop superior enzymes for weed, pathogen and pest control (Kababji et al., 2024). Biolistic delivery of ribonucleoprotein (RNP) complexes offers DNA-free editing while reducing off-target effects for generation of non-transgenic edited sugarcane events (Cai et al., 2025).

## Regulatory landscape and deployment

Argentina, Brazil, India, Australia and others have adopted permissive frameworks exempting certain gene-edited plants, which do not carry transgenes from stringent GMO regulations (Schmidt et al., 2020; Brant et al., 2025). Recent USDA regulatory amendments in the US (November 2024, subsequently vacated December 2024) illustrate evolving policy landscapes. The current “Am I Regulated” process favors biolistic transformation over Agrobacterium-mediated transfer. For commercial success, edited varieties must demonstrate substantial stress tolerance improvements without yielding penalties, maintain desirable sugar content, and show stable trait inheritance across vegetative propagation cycles.

## Discussion and future outlook

The convergence of CRISPR technology with sophisticated stress biology understanding positions sugarcane breeding at an inflection point. Demonstrated multiallelic editing feasibility coupled with successful field evaluation validates technical foundation (Brant et al., 2024a; Eid et al., 2021). Functional validation in sugarcane itself is essential, as Wang et al. (2025) exemplifies with *ScWRKY2*. Understanding gene regulatory networks through careful phenotypic evaluation under diverse conditions will prevent unintended consequences.

Integrating genome editing with conventional breeding offers the most promising path, leveraging both conventional selection for quantitative traits and precision editing for targeted improvements. Emerging technologies will accelerate progress. Long-read sequencing improves understanding of genome structure. Artificial intelligence predicts optimal edit sites and potential off-target effects. Advanced phenotyping technologies enable comprehensive field evaluation.

The development of stress-tolerant sugarcane through genome editing represents a necessary response to mounting challenges facing global agriculture. As evolving pathogens threaten productivity, the ability to rapidly introduce precise improvements will be essential for sustaining production supporting both food and energy security. The pioneering work of the past decade laid a solid foundation, and the next decade promises maturation and commercial-scale deployment, provided technical innovation continues and regulatory frameworks facilitate responsible innovation.
